# Supplementary material for: Bone disease imaging through the near-infrared-II window
Source: Nat Commun. 2023 Oct 9;14:6287. doi: 10.1038/s41467-023-42001-2 (PMC10562434; doi:10.1038/s41467-023-42001-2)
Supplement: Supplementary file 1 — Supplementary Information [file 41467_2023_42001_MOESM1_ESM.pdf]

Supplementary information

Bone Disease Imaging through the Near-Infrared-II Window

Chao Mi[1,2,3,4,#,*], Xun Zhang[1,#], Chengyu Yang[5], Jianqun Wu[5], Xinxin Chen[6], Chenguang Ma[1], Sitong Wu[1,2], Zhichao Yang[1,2], Pengzhen Qiao[1], Yang Liu[5], Weijie Wu[1], Zhiyong Guo[1,3,7], Jiayan Liao[2], Jiajia Zhou[2], Ming Guan[1,4], Chao Liang[6,*], Chao Liu[5,7,*], and Dayong Jin[1,2,*]

1. UTS-SUSTech Joint Research Centre for Biomedical Materials and Devices, Department of Biomedical Engineering, Southern University of Science and Technology, Shenzhen, China
2. Institute for Biomedical Materials and Devices (IBMD), Faculty of Science, University of Technology Sydney, Australia
3. National Institute of Extremely-Weak Magnetic Field Infrastructure, Hangzhou, China
4. Shenzhen Light Life Technology Co., Ltd., Shenzhen, China
5. Department of Biomedical Engineering, Southern University of Science and Technology, Shenzhen, China
6. Department of Biology, School of Life Sciences, Southern University of Science and Technology, Shenzhen, China
7. Guangdong Provincial Key Laboratory of Advanced Biomaterials, Southern University of Science and Technology, Shenzhen, China

*Corresponding author (email to: dayong.jin@uts.edu.au (D. Jin); liuc33@sustech.edu.cn (C. Liu); liangc@sustech.edu.cn (C. Liang); mic@sustech.edu.cn (C. Mi))

[#]These authors contributed equally to this work.

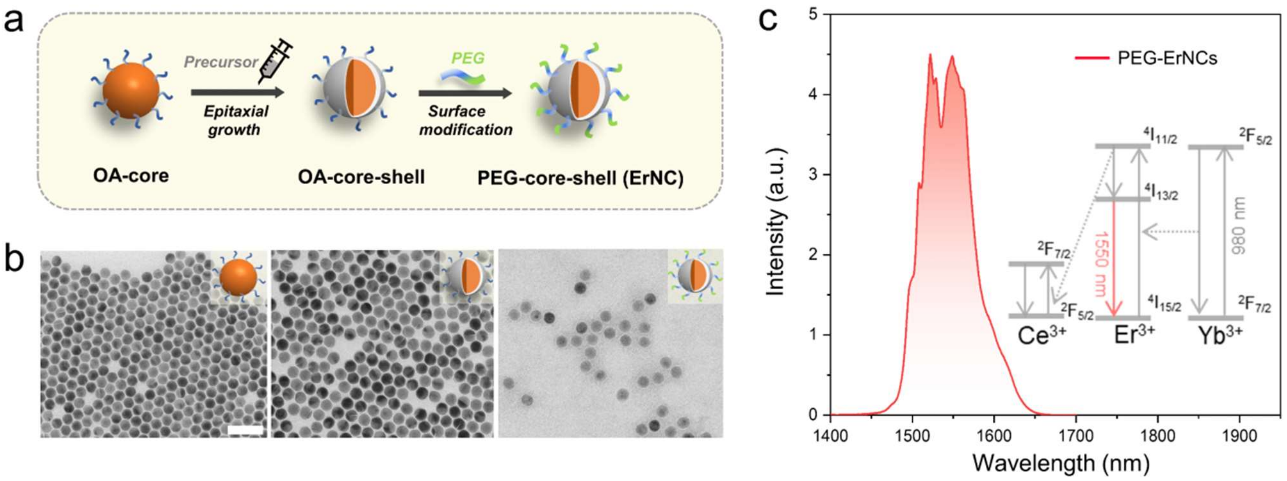

Figure S1. **a**, The preparation of 35 nm ErNCs. **b**, The TEM results of synthesized OA(olic acid)-capped core and core-shell ErNCs, and PEG-capped core-shell ErNCs from left to right, respectively. The doping concentration is 92% for Yb^{3+}, 4% for Er^{3+} and 4% for Ce^{3+}. Scale bar: 100 nm. **c**, The emission spectrum of the prepared PEG-capped core-shell structure ErNCs under a 980 nm continuous wave laser excitation. The insert illustrates the simplified energy transfer route among the doped Yb^{3+}, Er^{3+} and Ce^{3+} ions for ~1550 nm emission.

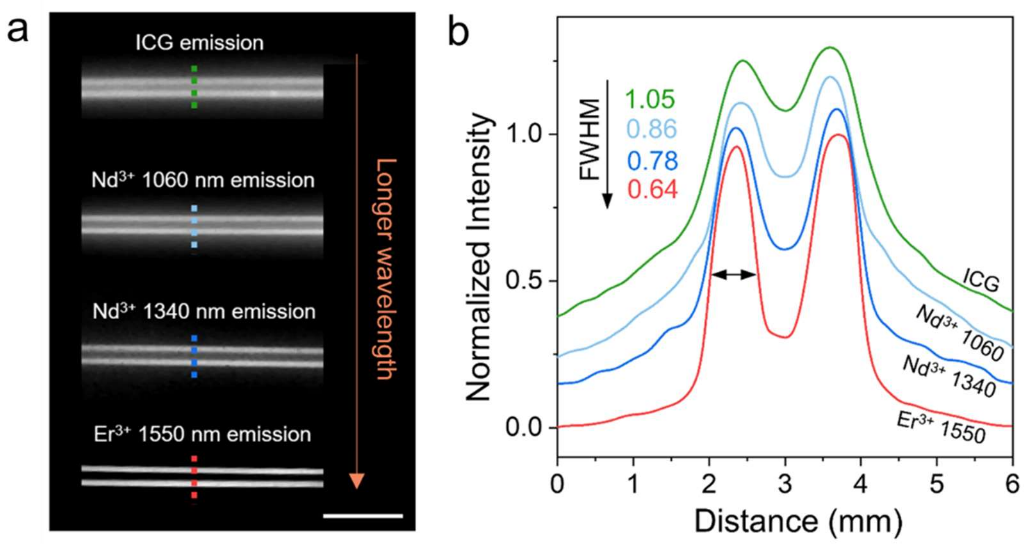

Figure S2. Comparison on the imaging resolution by different optical channels. **a**, The NIR-II fluorescence images of glass capillary pairs covered by the 2 mm thick 1% fat emulsion. The glass capillary pairs are filled by the clinically approved dye ICG (~1020 nm emission), NdNCs (~1064 nm emission and ~1340 nm emission respectively) and ErNCs (~1550 nm emission), respectively. Scale bar: 5 mm. **b**, Normalized signal intensity profiles and the full width at high maximum (FWHM) across the cross-sections indicated by dash lines in (a), respectively.

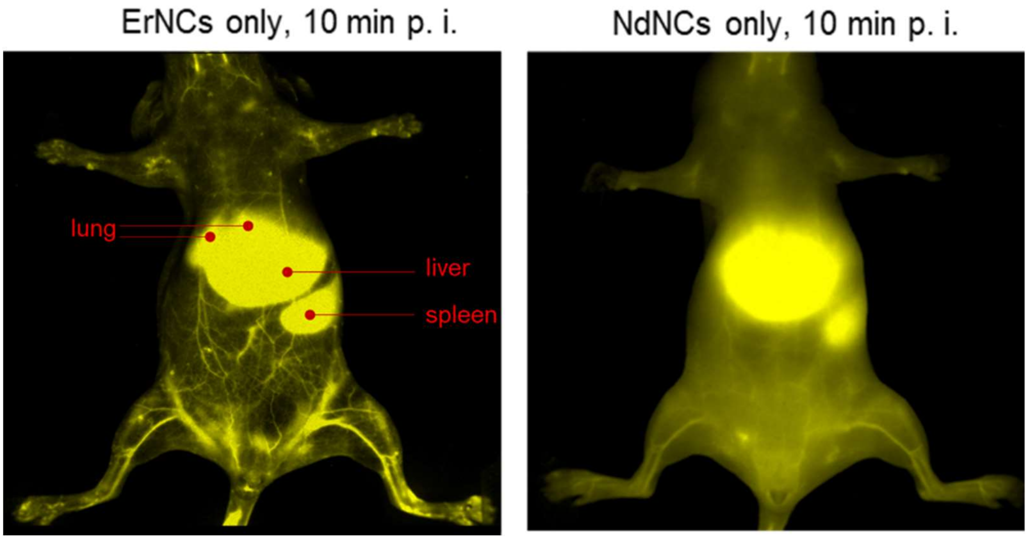

Figure S3. In vivo NIR-II imaging on mice blood vessel networks after 10 minutes (min) post injection (p. i.) with ErNCs of ~1550 nm emission (left), and NdNCs of ~1060 nm main emission band (right), respectively.

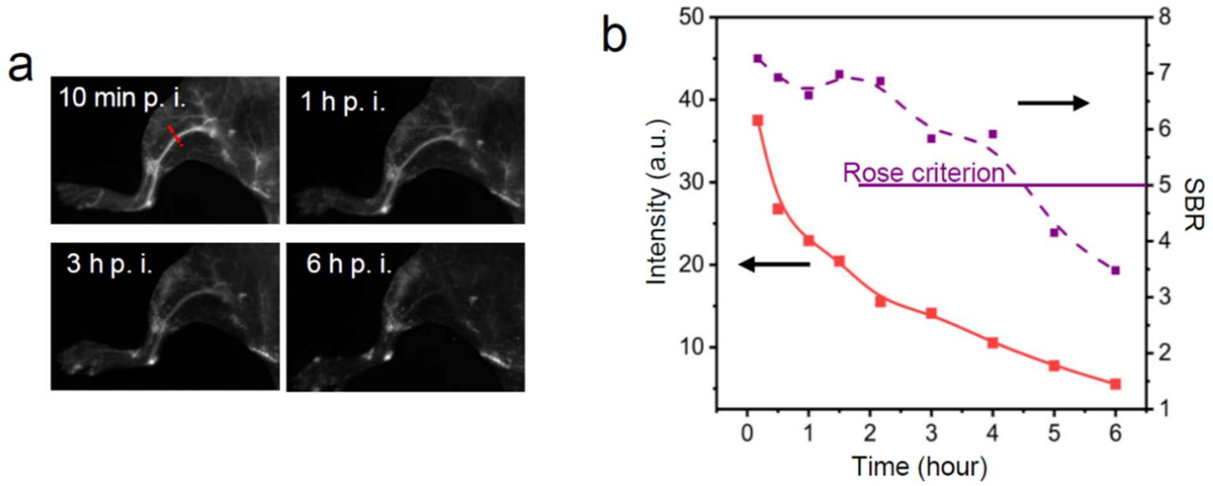

Figure S4. a, *In vivo* NIR-II imaging of blood vessel in mouse hindlimb at different time points after the injection of ErNCs (980 nm excitation, 38 mWcm^{-2}, 1319 nm long-pass, 60 ms). **b,** The time series of peak fluorescent intensity and signal to background ratio (SBR) along the same position represented by the red line in (a).

a b

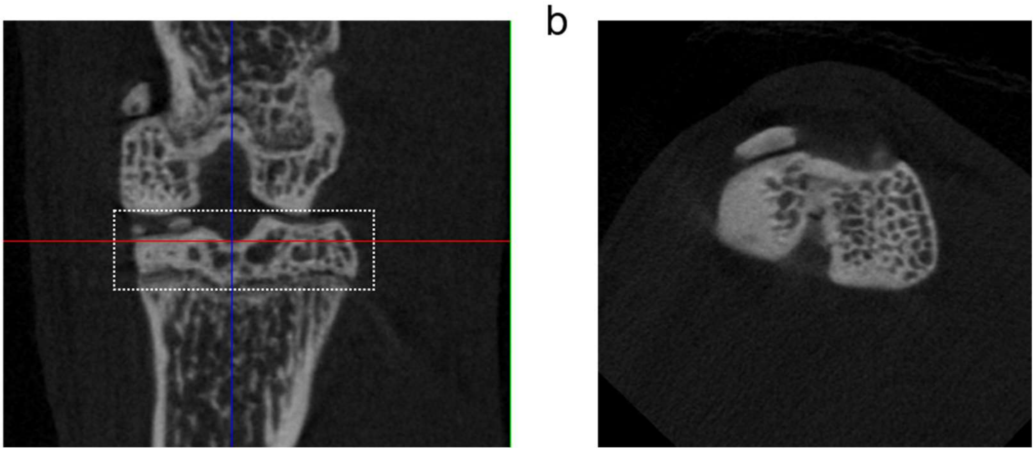

Figure S5. a, The subchondral bone in the dotted box confirmed by μCT. **b,** The cross-section view of the subchondral bone along the red line in (a).

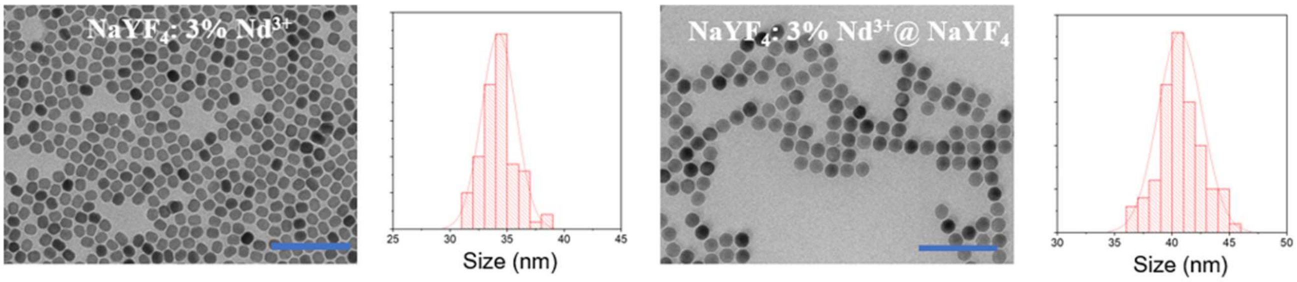

Figure S6. The TEM results and size distributions of synthesised $NaYF_4$: 3% Nd^{3+} and $NaYF_4$: 3% Nd^{3+}@ $NaYF_4$ (NdNCs) nanocrystals. Scale bars: 200 nm.

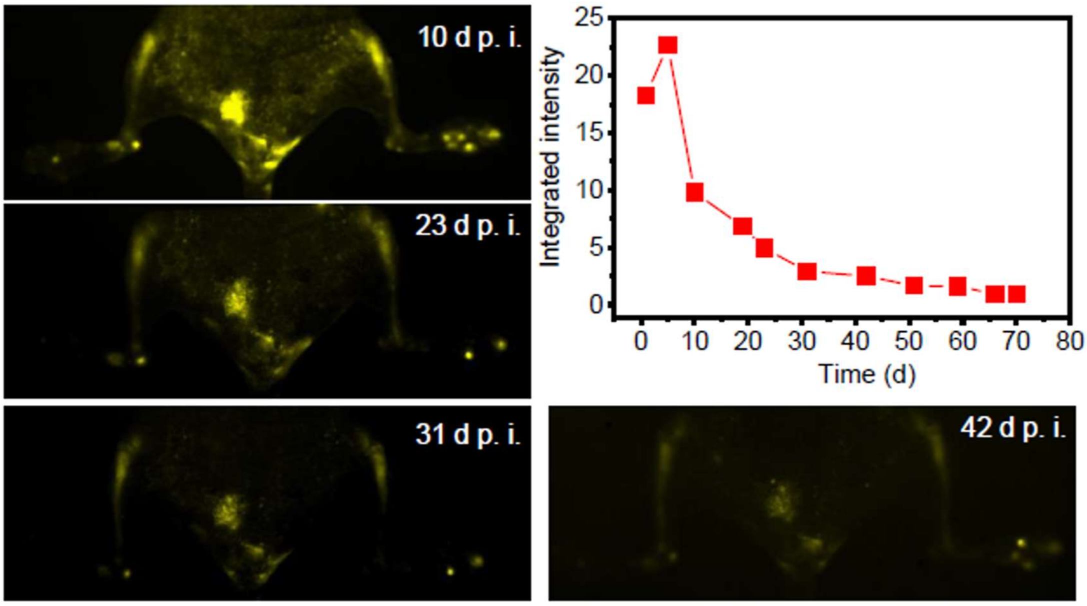

Figure S7. Long-term *in vivo* NIR-II imaging, and the fluorescent intensity change on mouse tibias from 1 to 71 d p. i. of ErNCs via tail vein (980 nm excitation, 38 mWcm^{-2}, 90 ms).

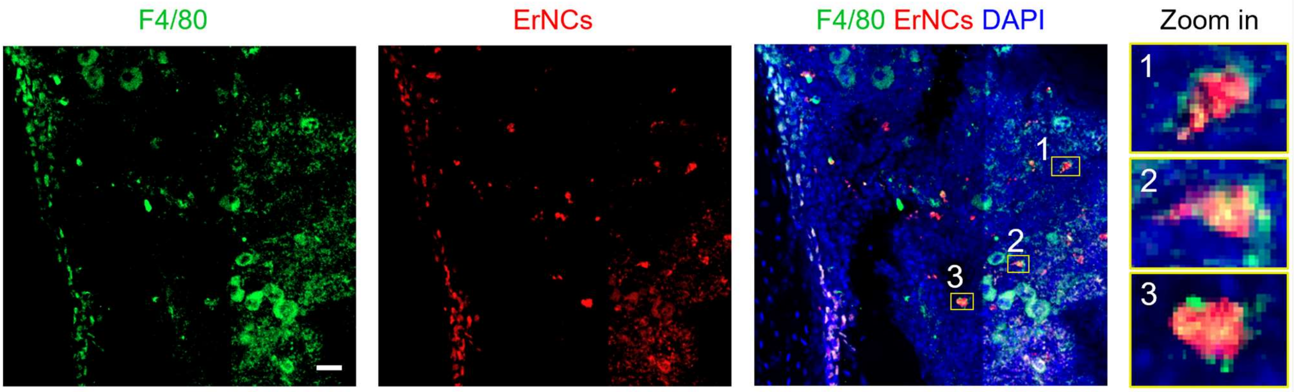

Figure S8. Confocal images of bone marrow in the stained tibia sections collected from a mouse 36 h after ErNCs@Cy3 injection, including three zoom-in regions of interest. Green channel: F4/80 labeled macrophages. Red channel: ErNCs@Cy3. Blue channel: DAPI labeled cell nucleus. Scale bar: 30 µm.

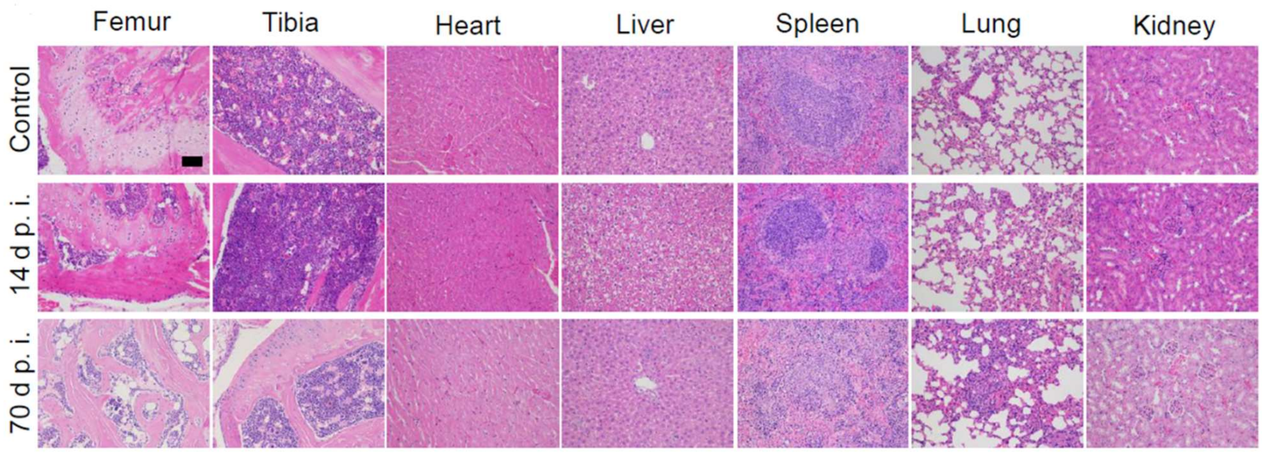

Figure S9. Hematoxylin and eosin (H&E) staining of the sectioned tissues (femur, tibia, heart, liver, spleen, lung, and kidney) from ErNCs treated mouse after 14 and 70 days of injection, and the PBS treated mouse as control. Scale bar: 100 μm.

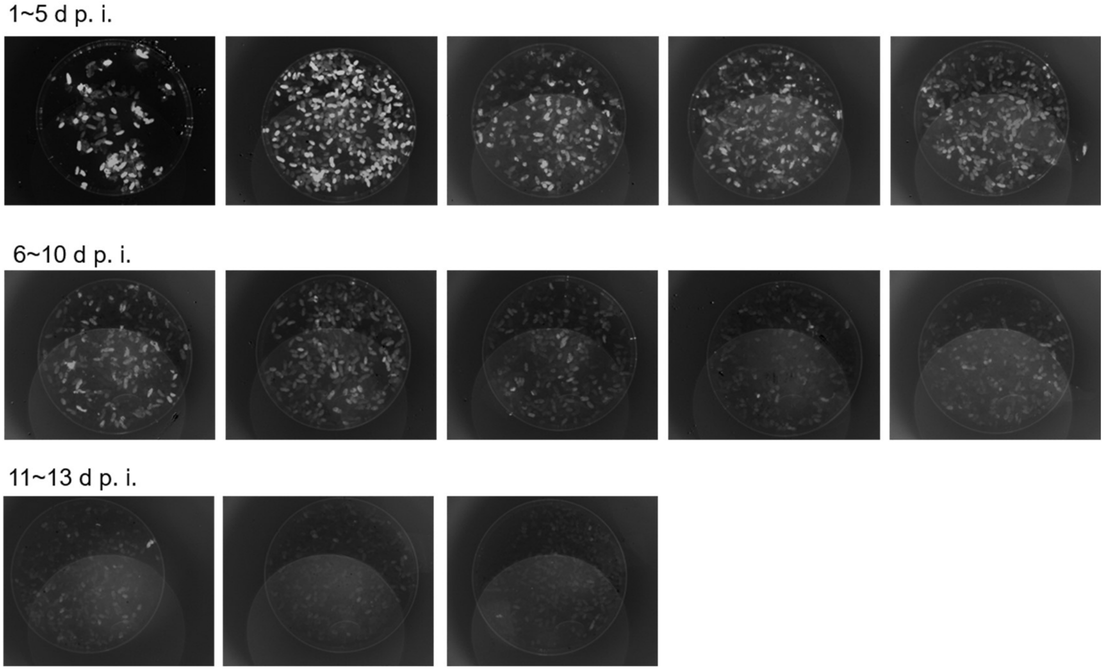

Figure S10. The long-term NIR-II imaging of all the feces excreted from the ErNCs-injected mice each day (980 nm excitation, 1 $mWcm^{-2}$, 1319 nm long-pass, 800 ms).

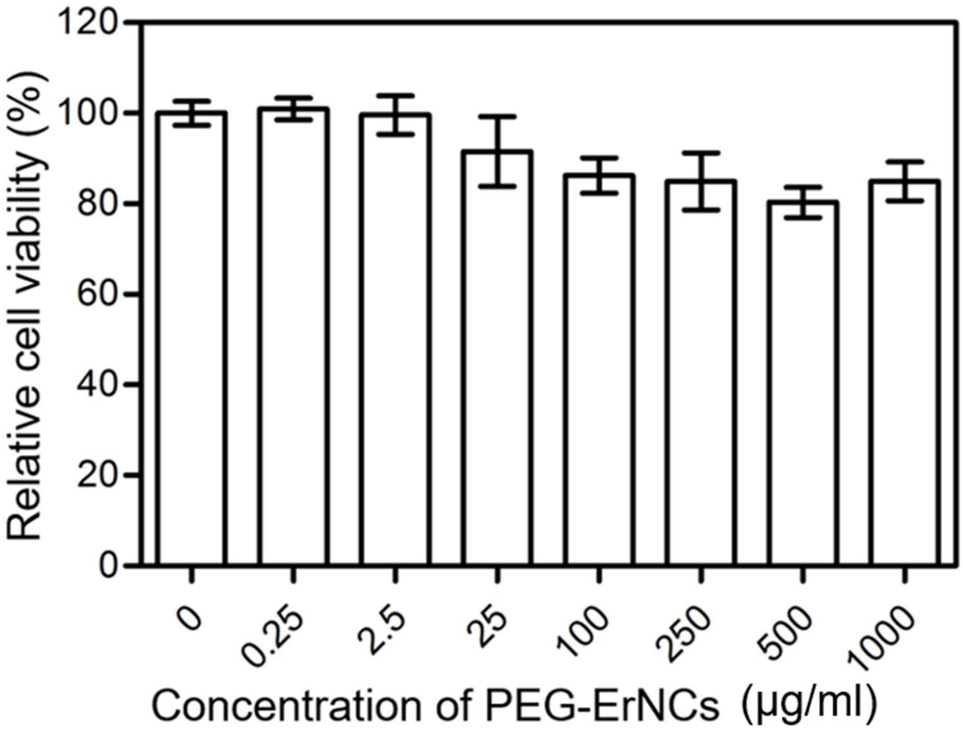

Figure S11. The cellular toxicity study of ErNCs in 4T1 cells.

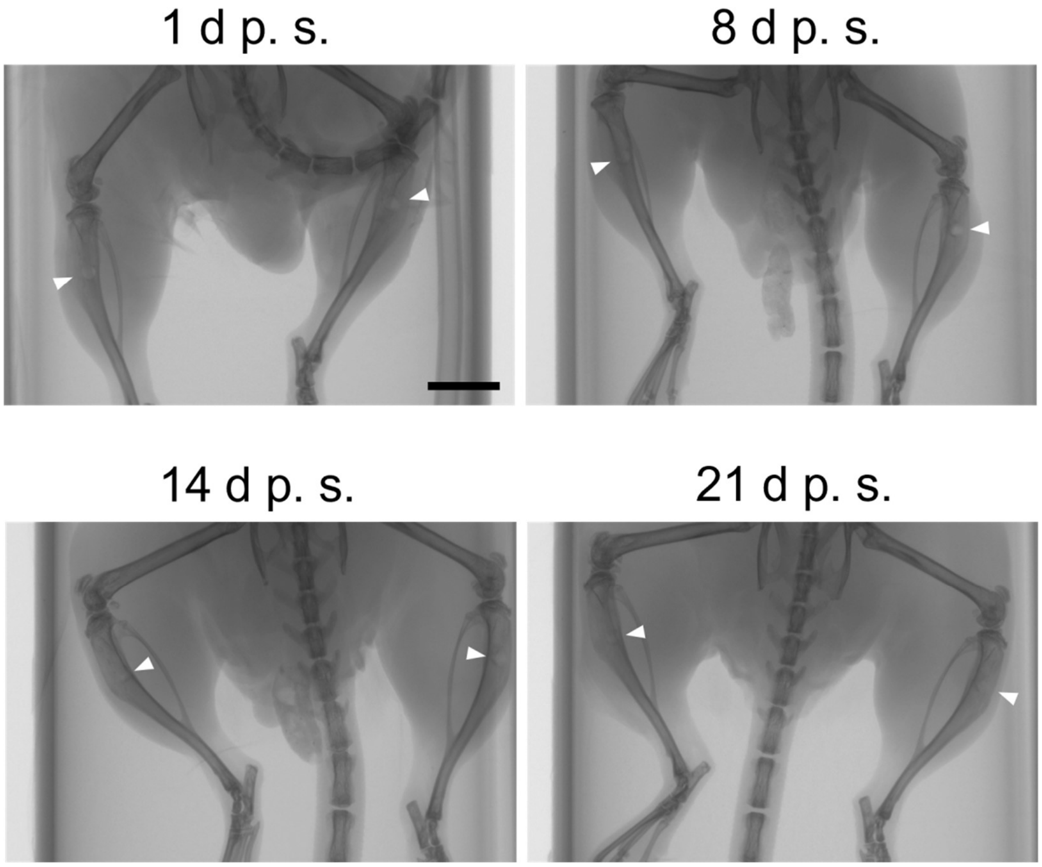

Figure S12. *In vivo* X-ray imaging on the ~1 mm tibia defects (arrowheads) on day 1, 8, 14 and 21 post surgery (p.s.). Scale bar: 5 mm.

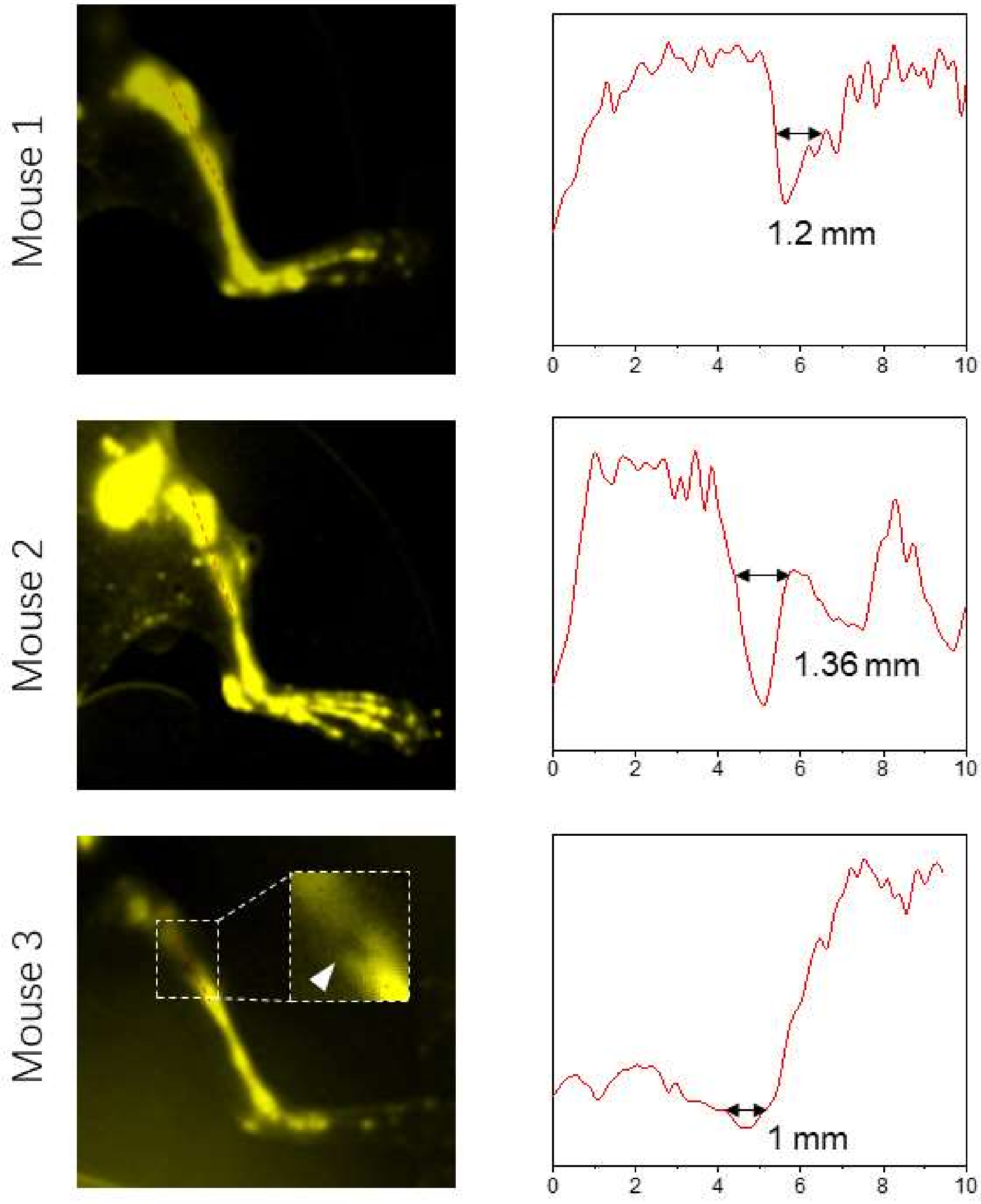

Figure S13. *In vivo* NIR-II imaging on bone defects of 3 mice. These results were carried out within 10 days of the bone surgery making on the left tibias of the mice.

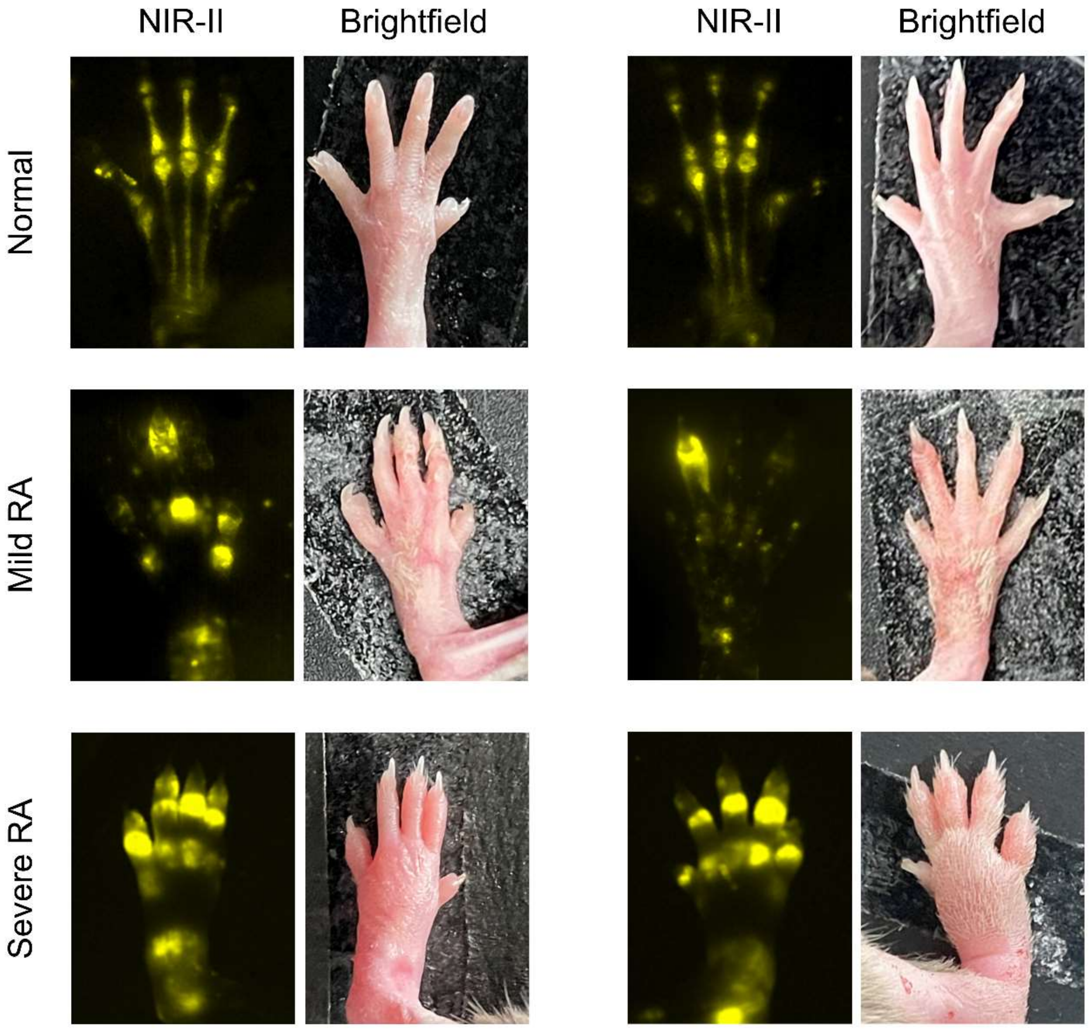

Figure S14. *In vivo* NIR-II and bright field imaging on the hind paws from normal mice, CIA model mice with mild RA, and the CIA model mice with severe RA, respectively.

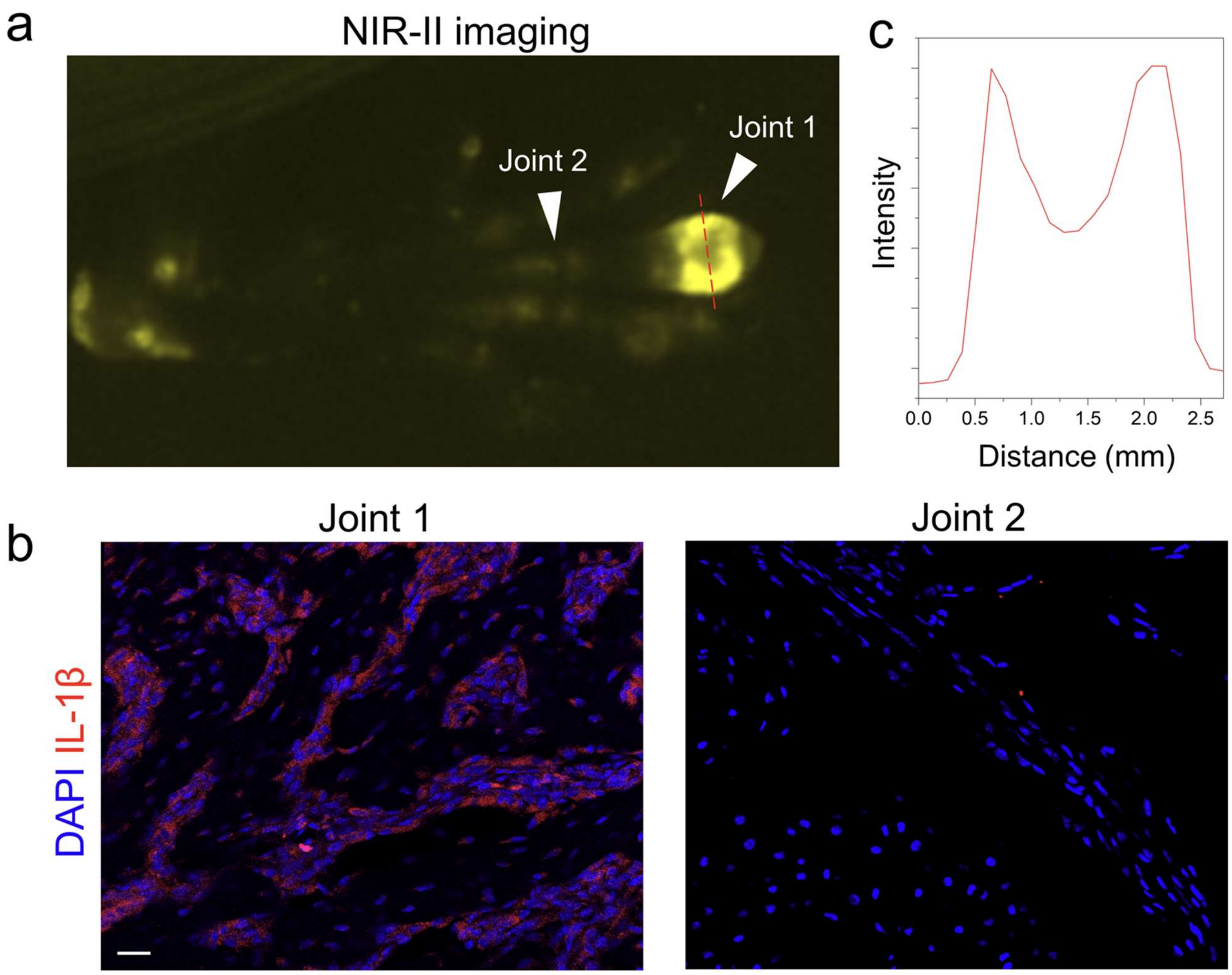

Figure S15. a, NIR-II imaging on the CIA mouse hind paw with early-stage arthritis. **b,** Immunofluorescence analyses of inflammation factor IL-1β in the synovium tissues from two toe joints (numbered arrowheads) in the hind paw in (a). Scale bar: 20 μm. **c,** The normalized intensity profiles along the cross section indicated by the dash line in (a).

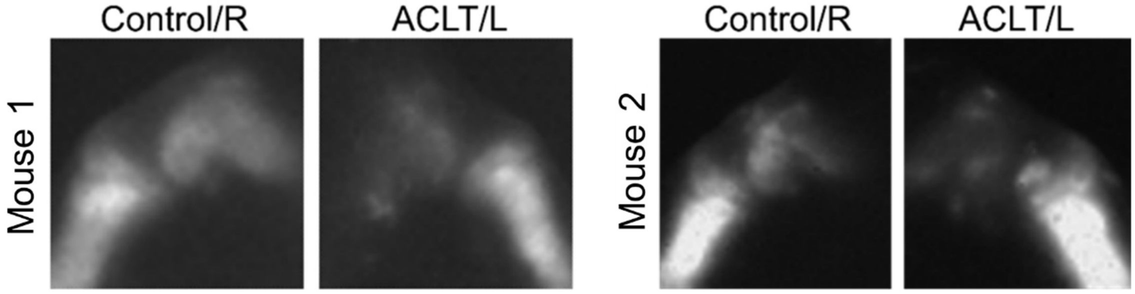

Figure S16. *In vivo* NIR-II imaging on the knee joints from two mice, the left knees of both Mouse 1 and Mouse 2 had an ACLT surgery 8 weeks ago, the right knees were untreated as control. (L: left, R: right)

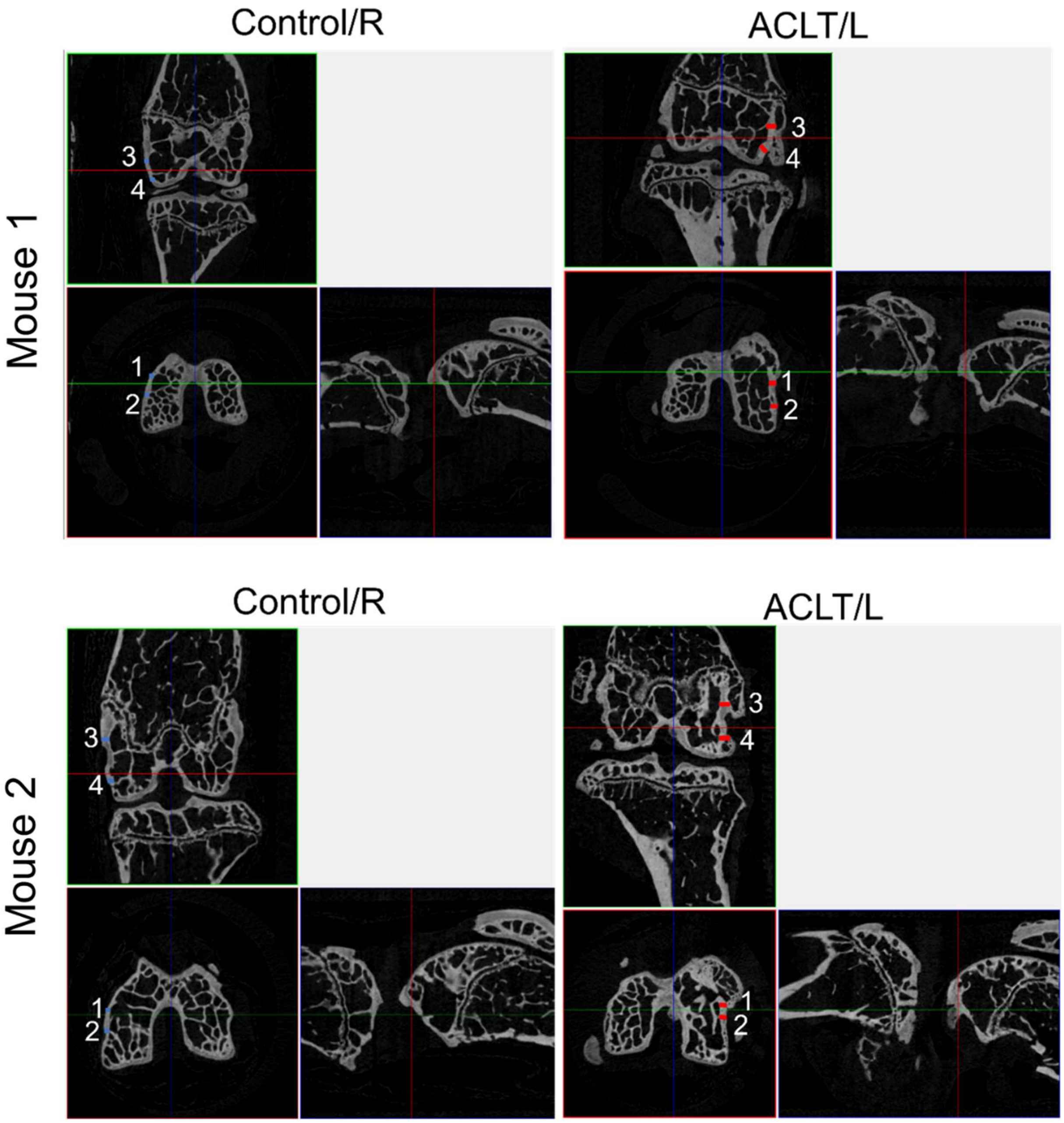

Figure S17. The *ex vivo* μCT cross-section images on the left and right femurs of Mouse 1 and 2 in Figure S13. The locations of the cross sections on the femurs are illustrated in Figure 5f.

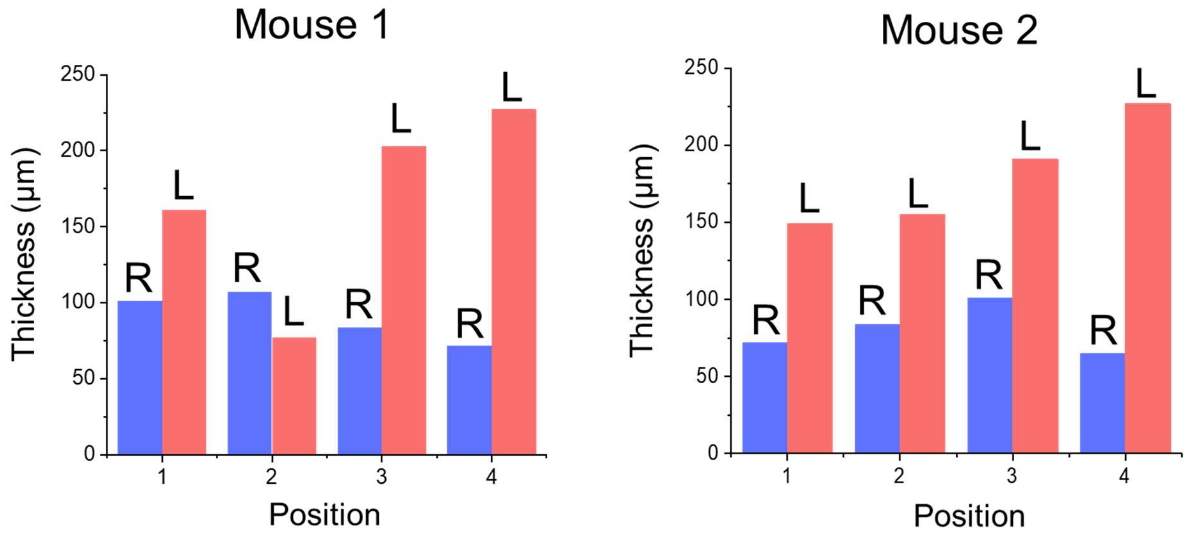

Figure S18. Comparison on the cortical bone thicknesses of 16 positions indicated by the numbered short lines (blue: Right femurs; red: Left femurs) in Figure S14. L: left, R: right.

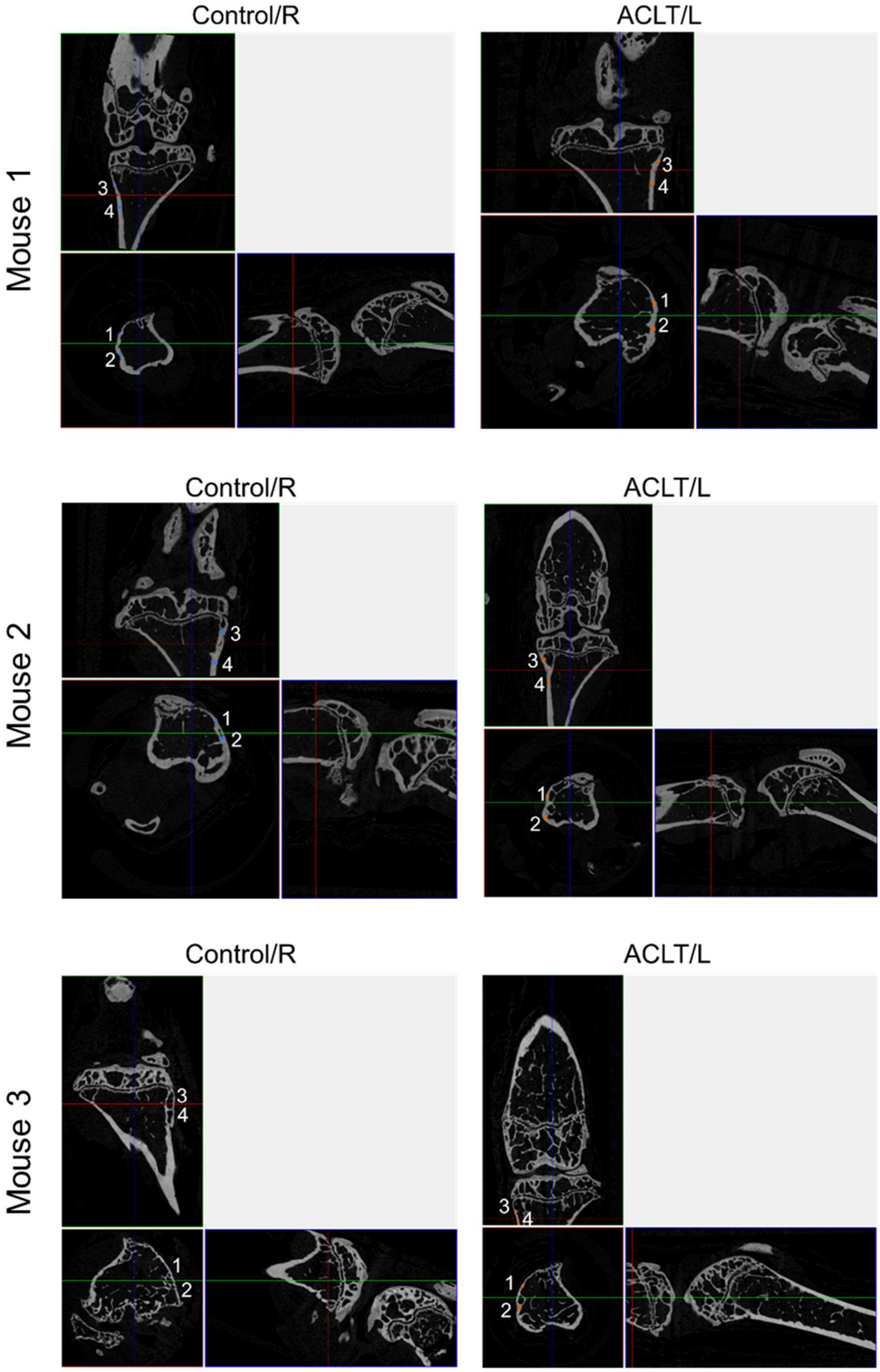

Figure S19. The *ex vivo* μCT cross-section images on the tibias of Mouse 1, 2 and 3. All the left knees had an ACLT surgery 8 weeks ago, and the right knees were untreated as control. L: left, R: right.

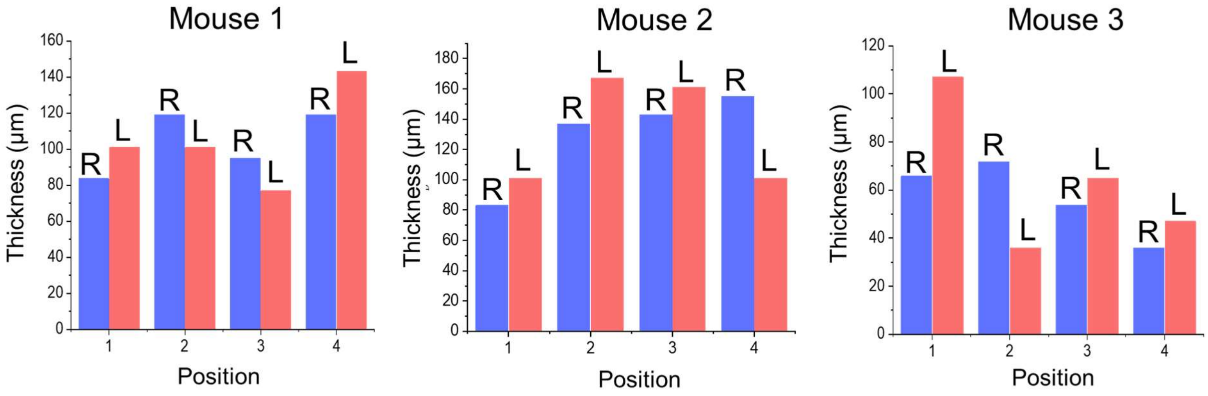

Figure S20. Comparison on the cortical bone thicknesses of 24 positions indicated by the numbered short lines (blue: Right tibias; red: Left tibias) in Figure S16 from all tibias of 3 mice.
