# Supplementary material for: Bone disease imaging through the near-infrared-II window
Source: Nat Commun. 2023 Oct 9;14:6287. doi: 10.1038/s41467-023-42001-2 (PMC10562434; doi:10.1038/s41467-023-42001-2)
Supplement: Supplementary file 3 — Description of Additional Supplementary Files [file 41467_2023_42001_MOESM3_ESM.pdf]

Description of Additional Supplementary Files

Supplementary Movie 1: In vivo 3D NIR-II mouse tibia imaging.

Supplementary Movie 2: Ex vivo 3D NIR-II mouse tibia imaging.
