# Supplementary material for: Bone disease imaging through the near-infrared-II window
Source: Nat Commun. 2023 Oct 9;14:6287. doi: 10.1038/s41467-023-42001-2 (PMC10562434; doi:10.1038/s41467-023-42001-2)
Supplement: Supplementary file 6 — Reporting Summary [file 41467_2023_42001_MOESM6_ESM.pdf]

nature portfolio

Corresponding author(s): Chao Mi, Chao Liang, Chao Liu, and Dayong Jin

Last updated by author(s): Aug 23, 2023

Reporting Summary

Nature Portfolio wishes to improve the reproducibility of the work that we publish. This form provides structure for consistency and transparency in reporting. For further information on Nature Portfolio policies, see our Editorial Policies and the Editorial Policy Checklist.

Statistics

For all statistical analyses, confirm that the following items are present in the figure legend, table legend, main text, or Methods section.

n/a	Confirmed	
☐	☒	The exact sample size (n) for each experimental group/condition, given as a discrete number and unit of measurement
☐	☒	A statement on whether measurements were taken from distinct samples or whether the same sample was measured repeatedly
☐	☒	The statistical test(s) used AND whether they are one- or two-sided<br>*Only common tests should be described solely by name; describe more complex techniques in the Methods section.*
☒	☐	A description of all covariates tested
☐	☒	A description of any assumptions or corrections, such as tests of normality and adjustment for multiple comparisons
☐	☒	A full description of the statistical parameters including central tendency (e.g. means) or other basic estimates (e.g. regression coefficient) AND variation (e.g. standard deviation) or associated estimates of uncertainty (e.g. confidence intervals)
☒	☐	For null hypothesis testing, the test statistic (e.g. F, t, r) with confidence intervals, effect sizes, degrees of freedom and P value noted<br>*Give P values as exact values whenever suitable.*
☒	☐	For Bayesian analysis, information on the choice of priors and Markov chain Monte Carlo settings
☒	☐	For hierarchical and complex designs, identification of the appropriate level for tests and full reporting of outcomes
☐	☒	Estimates of effect sizes (e.g. Cohen's d, Pearson's r), indicating how they were calculated

Our web collection on statistics for biologists contains articles on many of the points above.

Software and code

Policy information about availability of computer code

Data collection	ZolixScanBasic 2.0 (spectra), FirstLight Imagery 1.0(NIR-II imaging), PSLViewer 2.0(NIR-II imaging)
Data analysis	Imaris 9.5 (3D NIR-II imaging), Mimics 17 (CT), Origin 2018, MATLAB 2022a, ImageJ 1.52V

For manuscripts utilizing custom algorithms or software that are central to the research but not yet described in published literature, software must be made available to editors and reviewers. We strongly encourage code deposition in a community repository (e.g. GitHub). See the Nature Portfolio guidelines for submitting code & software for further information.

Data

Policy information about availability of data

All manuscripts must include a data availability statement. This statement should provide the following information, where applicable:

- Accession codes, unique identifiers, or web links for publicly available datasets
- A description of any restrictions on data availability
- For clinical datasets or third party data, please ensure that the statement adheres to our policy

The authors declare that other data related to this research are available within the paper and its Supplementary Information or from the authors upon request. Source data and codes are provided with this paper.

Research involving human participants, their data, or biological material

Policy information about studies with human participants or human data. See also policy information about sex, gender (identity/presentation), and sexual orientation and race, ethnicity and racism.

Reporting on sex and gender	N/A
Reporting on race, ethnicity, or other socially relevant groupings	N/A
Population characteristics	N/A
Recruitment	N/A
Ethics oversight	N/A

Note that full information on the approval of the study protocol must also be provided in the manuscript.

Field-specific reporting

Please select the one below that is the best fit for your research. If you are not sure, read the appropriate sections before making your selection.

[x] Life sciences [ ] Behavioural & social sciences [ ] Ecological, evolutionary & environmental sciences

For a reference copy of the document with all sections, see nature.com/documents/nr-reporting-summary-flat.pdf

Life sciences study design

All studies must disclose on these points even when the disclosure is negative.

Sample size	No statistical methods were used to predetermine sample sizes. Sample sizes for mice experiments were based on prior publications (Nat Biotechnol 37, 1322–1331, 2019) with suitable n numbers and a minimum of n of 3 animals for almost all of the mice experiments. The sample sizes meet a criterion of this field and can achieve statistical significance.
Data exclusions	No data were excluded from the analyses.
Replication	Number of successful replication experiment of the experimental findings is stated in the figure captions and methods
Randomization	Animal from different cage, but within the same experimental group, were selected to assure randomization. Nanoparticles from the different synthesize batches, but with same photo-physical properties, were allocated into experimental groups randomly
Blinding	The investigators were not blinded to group allocation during data collection, because the experiments and group allocation were completed by the same investigators.

Reporting for specific materials, systems and methods

We require information from authors about some types of materials, experimental systems and methods used in many studies. Here, indicate whether each material, system or method listed is relevant to your study. If you are not sure if a list item applies to your research, read the appropriate section before selecting a response.

Materials & experimental systems

n/a	Involved in the study	
[ ]	[x]	Antibodies
[ ]	[x]	Eukaryotic cell lines
[x]	[ ]	Palaeontology and archaeology
[ ]	[x]	Animals and other organisms
[x]	[ ]	Clinical data
[x]	[ ]	Dual use research of concern
[x]	[ ]	Plants

Methods

n/a	Involved in the study	
[x]	[ ]	ChIP-seq
[x]	[ ]	Flow cytometry
[x]	[ ]	MRI-based neuroimaging

Antibodies

Antibodies used	In the immunofluorescence text, tibia sections were stained with endomucin (sc-65495, Santa Cruz Biotechnology, USA), anti-F4/80 antibody (ab6640, Abcam, UK) followed by Alexa Fluor 488 secondary antibodies from donkey (A32790, Thermo Fisher Scientific, USA). Toe joint sections were stained with a rabbit anti-IL-1β (ab283818, Abcam, UK), and Alexa Flour 594 labeled goat anti-rabbit IgG

(ab150080, Abcam, UK) was used as the secondary antibody.

Validation	All the antibodies used were validated by the manufacturers. All the datasheets as the validations for the used antibodies can be find in the website of the Santa Cruz Biotechnology, Abcam, and Thermo Fisher Scientific according to the catalog number.

Eukaryotic cell lines

Policy information about cell lines and Sex and Gender in Research

Cell line source(s)	Mouse 4T1 cell line was provided by the Institute of Biochemistry and Cell Biology, SIBS, CAS (China).
Authentication	No authentication was performed.
Mycoplasma contamination	The cell line was ested negative for mycoplasma contamination.
Commonly misidentified lines (See ICLAC register)	The cell line used in this work is not included in the list of misidentified cell lines made by International Cell Line Authentication committee.

Animals and other research organisms

Policy information about studies involving animals; ARRIVE guidelines recommended for reporting animal research, and Sex and Gender in Research

Laboratory animals	All mice were purchased from Guangdong Medical Laboratory Animal Center (Guangdong, China), including 6-week-old male DBA/1 mice for collagen-induced arthritis (CIA) preparation and 6-week-old male BALB/c mice for other experiments. The mice were grown in an animal facility under filtered air conditions (21-22°C) in plastic cages with sterilized wood shavings for bedding and provided pure water. The mice were raised in a specific pathogen-free (SPF) environment.All animal experiments were strictly performed under the guidelines of the Chinese Council for Animal Care, approved by the Animal Care Committee of the Laboratory Animals in Southern University of Science and Technology.
Wild animals	This study dose not use wild animals.
Reporting on sex	Sex was not considered in the study design.
Field-collected samples	This study dose not involve samples collected from the field
Ethics oversight	All animal experiments in this work were strictly performed under the guidelines of the Chinese Council for Animal Care, approved by the Animal Care Committee of the Laboratory Animals in Southern University of Science and Technology.

Note that full information on the approval of the study protocol must also be provided in the manuscript.
